# AI task-shifting for echocardiographic LVEF assessment in Singapore: an economic evaluation

**DOI:** 10.1093/eschf/xvag069

**Published:** 2026-03-05

**Authors:** Aprajita Kaushik, Sameera Senanayake, Sanjeewa Kularatna, Khung-Keong Yeo, Nicholas Graves, Carolyn S P Lam, Huang Weiting, Chanchal Chandramouli, Jasper Tromp

**Affiliations:** Saw Swee Hock School of Public Health, National University of Singapore, 12 Science Drive 2, 117549 Singapore; Health Services Research & Population Health, Duke-NUS Medical School, 8 College Rd, 169857 Singapore; National Heart Research Institute Singapore, National Heart Centre, 5 Hospital Drive, 169609 Singapore; Australian Centre for Health Services Innovation (AusHSI), Queensland University of Technology, 60 Musk Avenue, Kelvin Grove, Brisbane, QLD 4059, Australia; Health Services Research & Population Health, Duke-NUS Medical School, 8 College Rd, 169857 Singapore; National Heart Research Institute Singapore, National Heart Centre, 5 Hospital Drive, 169609 Singapore; Australian Centre for Health Services Innovation (AusHSI), Queensland University of Technology, 60 Musk Avenue, Kelvin Grove, Brisbane, QLD 4059, Australia; Health Services Research & Population Health, Duke-NUS Medical School, 8 College Rd, 169857 Singapore; Health Services Research & Population Health, Duke-NUS Medical School, 8 College Rd, 169857 Singapore; Health Services Research & Population Health, Duke-NUS Medical School, 8 College Rd, 169857 Singapore; Australian Centre for Health Services Innovation (AusHSI), Queensland University of Technology, 60 Musk Avenue, Kelvin Grove, Brisbane, QLD 4059, Australia; Health Services Research & Population Health, Duke-NUS Medical School, 8 College Rd, 169857 Singapore; Australian Centre for Health Services Innovation (AusHSI), Queensland University of Technology, 60 Musk Avenue, Kelvin Grove, Brisbane, QLD 4059, Australia; Health Services Research & Population Health, Duke-NUS Medical School, 8 College Rd, 169857 Singapore; National Heart Research Institute Singapore, National Heart Centre, 5 Hospital Drive, 169609 Singapore; Saw Swee Hock School of Public Health, National University of Singapore, 12 Science Drive 2, 117549 Singapore; Australian Centre for Health Services Innovation (AusHSI), Queensland University of Technology, 60 Musk Avenue, Kelvin Grove, Brisbane, QLD 4059, Australia; Department of Cardiology, University Medical Centre Groningen, University of Groningen, Hanzeplein, 19713 GZ Groningen, The Netherlands

## Abstract

**Background:**

Accurate assessment of left ventricular ejection fraction (LVEF) is crucial for heart failure (HF) diagnosis but requires skilled sonographers. Artificial intelligence-enabled point-of-care (AI-POC) devices may enable novices to assess LVEF, potentially reducing healthcare costs. We conducted a cost-minimization analysis comparing conventional sonographer-performed echocardiography versus novice-operated AI-POC devices.

**Methods:**

Using a decision tree model, we compared the costs of diagnosing LVEF <50% in patients with suspected heart failure across two pathways: novice-operated AI-POC devices versus standard transthoracic echocardiogram (TTE) performed by sonographers. The model incorporated LVEF <50% prevalence, diagnostic accuracy metrics, and comprehensive cost data for both approaches. We conducted a probabilistic sensitivity analysis to test the robustness of our findings under varying assumptions.

**Results:**

The AI-POC pathway demonstrated substantial cost savings, averaging S$1185 [US$1422] per patient compared to S$1403 [US$1684] for conventional TTE. In a single tertiary referral centre in Singapore, implementing AI-POC devices for LVEF assessment in 100 patients resulted in savings of S$21 669 [US$26 013]. Probabilistic sensitivity analysis suggested a 99.9% probability that the AI-POC approach would be cost-saving compared to standard TTE.

**Conclusions:**

This study provides economic evidence that task-shifting echocardiographic assessment of LVEF to novices using AI-POC devices is likely cost-saving compared to standard TTE. This task-shifting strategy offers a cost-saving alternative to conventional sonographer-led TTE.

## Background

Heart failure (HF) is associated with an increased risk of hospitalization with significant costs to the health system.^1^ In elderly inpatients, HF is the most common diagnosis. In Singapore, which has among the oldest population globally, the prevalence of HF is estimated at 4.5%.^2^ With accelerated ageing and improved treatment outcomes of patients with HF, the prevalence of HF in Singapore is expected to increase.

The financial costs of HF to the health system are significant.^3^ In high-income settings, managing HF accounts for 1% to 2% of total healthcare expenditures.^3^ Over 60% of HF health expenditures are direct medical costs, including physician costs, echocardiography, and medication.^4^ A previous analysis from Singapore showed that inpatient costs for patients with HF are substantial and accelerate in patients later in life.^5^ Therefore, early diagnosis is key to starting treatment early, which reduces the risk of HF hospitalization and is cost-effective.^6^

Echocardiography is the cornerstone of HF diagnosis due to a reduced left ventricular ejection fraction (LVEF). Unfortunately, a lack of trained sonographers causes long waiting times, delaying diagnosis and initiating cost-effective treatments.^7,8^ Artificial intelligence (AI)-supported point-of-care ultrasound (AI-POCUS) can empower novices with limited training to acquire high-quality images and assess LVEF with similar accuracy to human experts.^9–11^ However, the potential differences in costs of novice-led AI-POCUS versus a conventional sonographer-led transthoracic echocardiographic exam (TTE) remain unclear.

## Aim

This study analysed the potential cost savings associated with task-shifting echocardiographic LVEF assessment from sonographers to novices using an AI-POC device compared to a sonographer-led TTE from Singapore’s health systems perspective.

## Methods

### Study design

In our previous study, AI-supported interpretation of echocardiographic images was equally effective compared to human expert-interpreted images.^9^ Therefore, the proportion of patients correctly diagnosed with a reduced LVEF and started on treatment for HF with reduced ejection fraction (HFrEF) did not statistically differ in our current analysis. As a result, we compared cost differences between a novice-led AI-POC pathway and a conventional sonographer-led TTE using cost minimization analysis. For each model, we included all costs across groups until groups were equivalent (i.e. until patients were diagnosed with an LVEF <50% or not), consistent with best practices.

### Model overview

We developed a decision tree model using TreeAge Pro 2024 R1.1 (TreeAge Software, Williamstown, MA, USA) to compare the costs of diagnosing an LVEF <50% by novices using the AI-POC device against sonographers using a standard TTE. We considered all costs from a health systems perspective using a 1-year time horizon. We assumed (i) the patients could be hospitalized once within 12 months; and (ii) patients diagnosed with HF were receiving quadruple treatment therapy, i.e. Sodium-Glucose Cotransporter-2 Inhibitors (SGLT2i), Angiotensin Converting Enzyme (ACE-I) Inhibitor or Angiotensin-2 Receptor Blocker (ACE/ARB), Mineralocorticoid Receptor Antagonist (MRA), and Beta-adrenergic blockers (BB) [SGLT2i + ACEi/ARB + MRA + BB].^12^

The decision tree had two arms: the novice-led AI-POCUS arm and a sonographer-led TTE arm. The novice-led AI-POCUS arm averaged the costs of POCUS devices, including the Kosmos Torso-One, Philips Lumify, GE Vscan Air SL, or Butterfly iQ3+. We considered a previously validated deep learning workflow for the AI software that automatically classifies images and annotates LVEF (Us2.ai, Singapore).^13^ This workflow has previously been successfully used to screen for a reduced LVEF in combination with a POCUS device in an in-patient setting in Singapore^11^ and an outpatient setting in Tunisia.^10^ This was compared to care-as-usual, which in Singapore consists of a standard TTE performed and interpreted by a trained cardiac sonographer.

Model parameters included LVEF <50% prevalence rate, risk of HF hospitalization on and off guideline-directed medication, costs of each arm, and diagnostic performance (sensitivity and specificity) of the two arms. When patients were diagnosed, we considered patients to be on all recommended HF medications, including ACEi/ARBs, beta-blockers, MRAs, and SGLT2 inhibitors, as per current ESC guidelines for diagnosis and treatment of HF. The primary outcome was patient-level costs for each of the two arms, and all costs were considered until patients were correctly diagnosed. We simulated a hypothetical cohort of 100 patients through the two arms. A probabilistic sensitivity analysis (PSA) assessed uncertainties across the full range of model parameters and assumptions, with Monte Carlo simulations with 1000 iterations.

### Model inputs

*Table 1* shows the model inputs with uncertainty estimates and their sources. Costs were obtained from previously published data or local resources.^14^ The costs of medications were derived from the Singapore General Hospital formulary. A micro-costing approach was used to calculate the total cost per echocardiography for both the AI-led intervention (Us2ai) and the comparator (TTE). The total cost per patient included the unit cost per echocardiography (derived by dividing the total annual cost of equipment and software by the average number of outpatient echocardiographies conducted annually at the study site) and personnel costs (derived by calculating a salary-derived per-minute rate and multiplying it by the average time required per echocardiography for the respective healthcare provider (nurse, sonographer, or doctor)). This method ensured a standardized cost estimation framework across both approaches. Data on LVEF <50% prevalence was obtained from a recent inpatient study.^11^ The sensitivity and specificity of the intervention and manual TTE were obtained from a recent prospective study using the same deep-learning workflow.^9^ The probability of hospitalization within 1 year of treatment was obtained from SingCLOUD—a data bank from NHCS and its participating institutions (MOH, all restructured hospitals and polyclinics). All costs are in 2023 SG dollars, converted to 2023 US dollars, using the ‘CCEMG-EPPI Centre Cost Converter’ web-based tool.

**Table 1:** Parameter estimates used in the model and sensitivity analysis

Parameter	Estimate	Distribution	Source
Cardiologist outpatient consultation	$145 [US$174]	Gamma (127, 162) [US$152 665]	NHCS
Cost of hospitalisation	$9074 [US$10 893]	Gamma (1586, 33 709) [US$1903; 40 466]	^14^
Cost of Novice-led AI-POC device^a^	$12.46 [US$14.96]	Gamma (2.52, 25.76) [US$3.03; 30.92]	NHCS
Cost of TTE^a^	$33.98 [US$40.79]	Gamma (19.90, 52.57) [US$23.89; 63.11]	NHCS
Cost of treatment (monthly) (SGLT2i + ACE/ARB + BB + MRA)	$158.78 [US$190.61]	Gamma (90.51, 227.04) [US$108.66; 272.56]	SGH formulary
Novice-led AI-POC device			
*Sensitivity (True positive rate)*	*0*.*91*	Beta (0.88, 0.93)	^9^
*Specificity (True negative rate)*	*0*.*92*	Beta (0.87, 0.95)	^9^
*Probability of retrieving interpretable images (visually understandable AI images)*	*0*.*96*	Beta (0.86, 1.00)	PANES-HF trial
TTE			
*Sensitivity (True positive rate)*	*0*.*92*	Beta (0.89, 0.93)	^9^
*Specificity (True negative rate)*	*0*.*78*	Beta (0.74, 0.85)	^9^
Probability of hospitalisation due to HF within 1 year			
*With treatment (patients on quadruple therapy)*	*0*.*17*	Beta (0.13, 0.22)	^SingCLOUD^
*Without treatment (patients with no therapy)*	*0*.*19*	Beta (0.15, 0.24)	SingCLOUD

AI-POC, artificial intelligence-supported point-of-care device; NHCS, National Heart Centre Singapore; SGH, Singapore General Hospital; TTE, transthoracic echocardiography; SGLT2i, Sodium-Glucose Cotransporter-2 Inhibitors; ACE, Angiotensin Converting Enzyme (ACE-I) Inhibitor; ARB, Angiotensin-2 Receptor Blocker; BB, Beta-adrenergic blockers; MRA, Mineralocorticoid Receptor Antagonist.

^a^Are inclusive of personnel training, salary, maintenance and other overhead costs.

## Results

**Figure 1:**
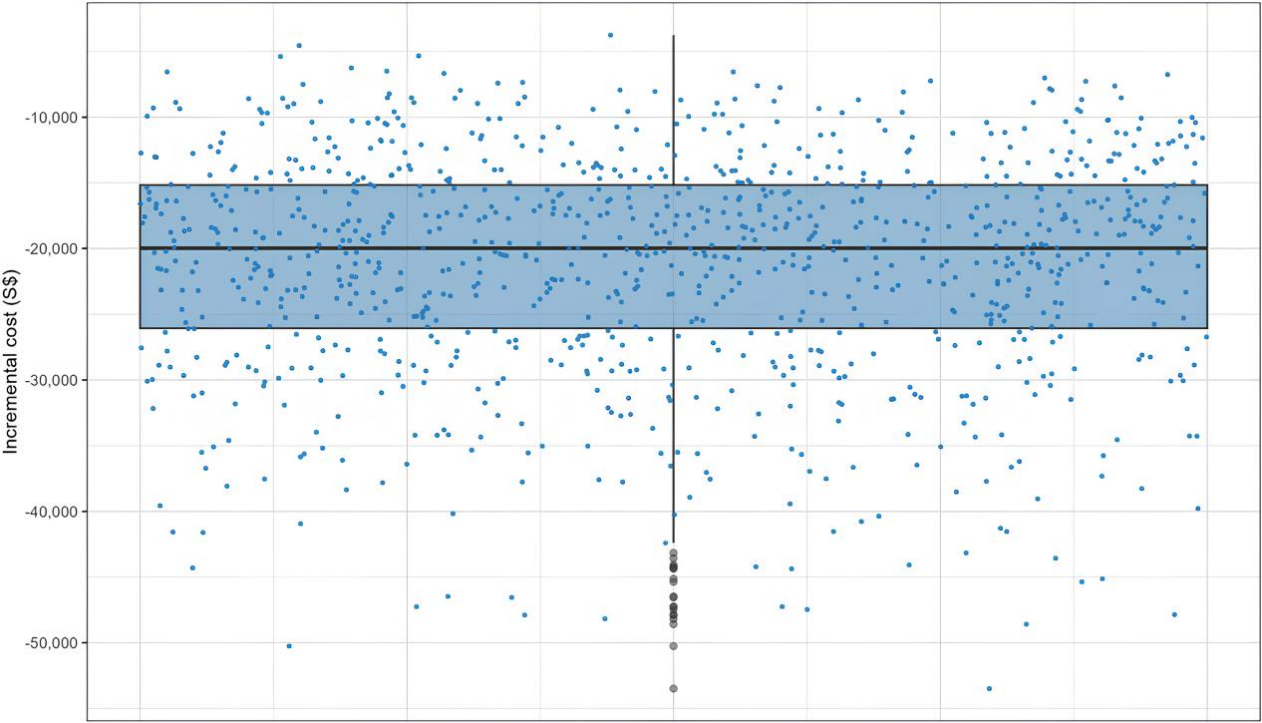
Incremental expected values for the novice-led artificial intelligence-enabled point-of-care diagnostic pathway compared to the standard transthoracic echocardiogram. Each dot indicates the incremental cost of replications generated from the 1000 iterations in the probabilistic sensitivity analysis

*Table 2* shows that the novice-led AI-POC pathway ($1175 [US$1410] per patient) is cost-saving compared to the standard TTE ($1387 [US$1665] per patient). According to PSA, the average cost for 100 patients undergoing novice-led AI-POC pathway was $118 592 [US$142 367]. In contrast, the standard TTE cost for the same number of patients was $140 261 [US$168 380], indicating a cost-saving of $21 669 [US$26 013] when opting for the novice-led AI-POC pathway compared to the sonographer-led TTE (*Figure 1*). PSA demonstrated that the probability of a novice-led AI-POC pathway being cost-saving was 99.9%.

**Table 2:** Cost savings of novice-led artificial intelligence-supported point-of-care device against sonographer-led transthoracic echocardiography

Strategy	Base case analysis	Probability sensitivity analysis Mean (95% CI)	Incremental value Mean (95% CI)	Probability of cost saving
*TTE*	$138 663 [US$166 462]	$140 261 [US$168 380] (136 056; 144 465) [US$163 332; 173 427]		
Intervention^a^	$117 518 [US$141 078]	$118 592 [US$142 367] (114 563; 122 621) [US$137 530; 147 204]	−$21 669 [−US$26 013] (−21 493, −21 845) [−US$25 801; 26 224]	99.9%

^a^. Novice-led artificial intelligence supported point of care ultrasound (POCUS).Results are presented for 100 patients for 1-year time horizon.

## Conclusion

Our study showed that novice-led AI-POCUS-supported assessment of LVEF in patients suspected of a reduced LVEF was cost-saving compared to care as usual. Previous studies found that deep learning-based assessments of LVEF were interchangeable with human expert assessments.^9,15^ Recently presented data showed that AI assistance can reduce examination time, reduce sonographers’ mental fatigue, and increase the number of daily studies analysed.^9,10,15^ Our results extend these previous findings by showing that deep learning-supported novice-led AI-POCUS is cost-saving compared to conventional TTE. To our knowledge, this is the first study to compare cost savings between a novice-led AI-POCUS-supported assessment of LVEF and a conventional sonographer-led TTE in an in-hospital setting. However, our results are not generalizable, and further assessment, supplemented with local costs and epidemiological data, is needed to validate the findings in other contexts.

In conclusion, our results suggest that novice-led AI-POCUS echocardiography might lead to time and cost savings compared to care as usual in Singapore. Similar task-shifting approaches can help reduce the burden on echocardiographic labs, reduce resource utilization, and reduce time to diagnosis.

## Declarations

### Data Availability

No data were generated or analysed for this manuscript.
